# Embryonic neural stem/progenitor cells as model to characterize dystrophin and dystrophin-associated proteins expression during neuronal or astrocytic differentiation

**DOI:** 10.1016/j.mex.2021.101325

**Published:** 2021-03-27

**Authors:** José Romo-Yáñez, Griselda Rodríguez-Martínez, Jorge Aragón, Lourdes Siqueiros-Márquez, Alma Herrera-Salazar, Iván Velasco, Cecilia Montanez

**Affiliations:** a Departamento de Genética y Biología Molecular, Centro de Investigación y de Estudios Avanzados (Cinvestav), Ciudad de México, Mexico; b Present address: Coordinación de Endocrinología Ginecológica y Perinatal, Instituto Nacional de Perinatología, Ciudad de México, Mexico; c Instituto de Fisiología Celular-Neurociencias, Universidad Nacional Autónoma de México (UNAM), Ciudad de México, Mexico; d Laboratorio de Reprogramación Celular, Instituto Nacional de Neurología y Neurocirugía, Ciudad de México, Mexico

**Keywords:** Embryonic neural stem/progenitor cells, Neuronal and glial differentiation, Dystrophin and dystrophin-associated proteins

## Abstract

Neural stem/progenitor cells (NSPC) are multipotent cells that renew themselves and could differentiate into neurons and macro glia (astrocytes and oligodendrocytes) of the nervous system during embryonic development. Duchenne muscular dystrophy is a severe type of muscular dystrophy caused by mutations in the *dmd* gene, and one-third of patients cursed with neuro-cognitive impairments. In this data article, we take advantage of the differentiation capacity of NSPC as a model to increase our knowledge in the neuronal and/or astrocytic differentiation and to evaluate the expression of dystrophins and dystrophin-associated proteins. We showed the characterization of undifferentiated and neuron and/or astrocyte differentiated NSPC. In addition, we evaluated the expression and subcellular localization of dystrophins and β-dystroglycan in undifferentiated NSPC and differentiated to neurons and astrocytes.

• Primary culture of NSPC was characterized by the expression of multipotent markers nestin and Sox2.
• Neuronal or astrocytic differentiation of NSPC was performed by basic fibroblast growth factor (FGF2) withdrawal, histamine or ciliary neurotrophic factor (CNTF) treatment, and expression of βIII-tubulin or glial fibrillary acidic protein (GFAP) as differentiation markers for neurons or astrocytes was evaluated.
• This study will contribute to the understanding of dystrophins and dystrophin-associated proteins expression and function during neuronal or astrocytic differentiation of NSPC

## Graphical abstract

**Figure:**
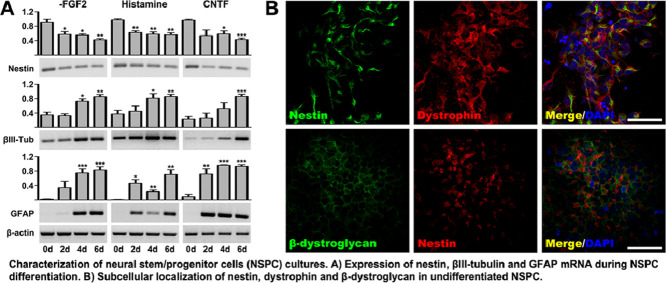

Specifications table

**Table:** 

Subject Area:	Neuroscience
More specific subject area:	Molecular biology
Method name:	Isolation and culture of embryonic neural stem/progenitor cells
Name and reference of original method:	G. Rodríguez-Martínez, A. Molina-Hernández, I. Velasco, Activin A promotes neuronal differentiation of cerebrocortical neural progenitor cells, PloS One 7 (2012) e43797. A. Molina‐Hernández, I. Velasco, Histamine induces neural stem cell proliferation and neuronal differentiation by activation of distinct histamine receptors, J. Neurochem. 106 (2008) 706–717. C. Sauvageot, P.L. Dahia, O. Lipan, J.K. Park, M.S. Chang, J.A. Alberta, C.D. Stiles, Distinct temporal genetic signatures of neurogenic and gliogenic cues in cortical stem cell cultures, J. Neurobiol. 62 (2005) 121–133.
Resource availability:	All data are presented in this article.

## Background

**Fig. 1:**
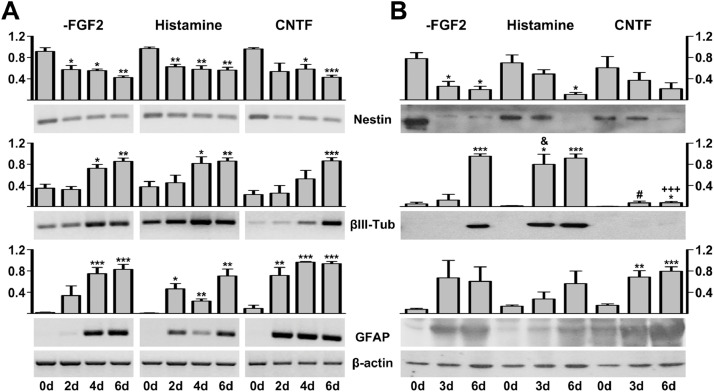
Expression of nestin, βIII-tubulin and GFAP during NSPC differentiation. Total RNA and protein were isolated from passage 2 embryonic NSPC either during proliferation or at distinct differentiation stages after FGF2 withdrawal (-FGF2) or after treatment with histamine or CNTF. (A) RT-PCR showing nestin, βIII-tubulin and GFAP mRNA. (B) Western blot showing nestin (pAb), βIII-tubulin (mAb) and GFAP (pAb) proteins. Nestin down-regulation was used as a differentiation control. βIII-tubulin (βIII-Tub) and GFAP expression were detected as neuron and astrocyte markers, respectively. β-actin mRNA and protein were used as loading controls in the RT-PCR and western blot assays, respectively. Lanes: 0d (undifferentiated), 2d, 3d, 4d and 6d (time-points indicating the days of differentiation). Graphs show the mean plus standard error of three independent experiments. *P < 0.05, **P < 0.01 and ***P < 0.001 denote statistical significance versus 0d of each treatment; ^&^*P* < 0.05 denotes statistical significance versus –FGF2 3d; ^#^*P* < 0.05 denotes statistical significance versus histamine 3d; ^+++^*P* < 0.001 denotes statistical significance versus -FGF2 and histamine 6d.

**Fig. 2:**
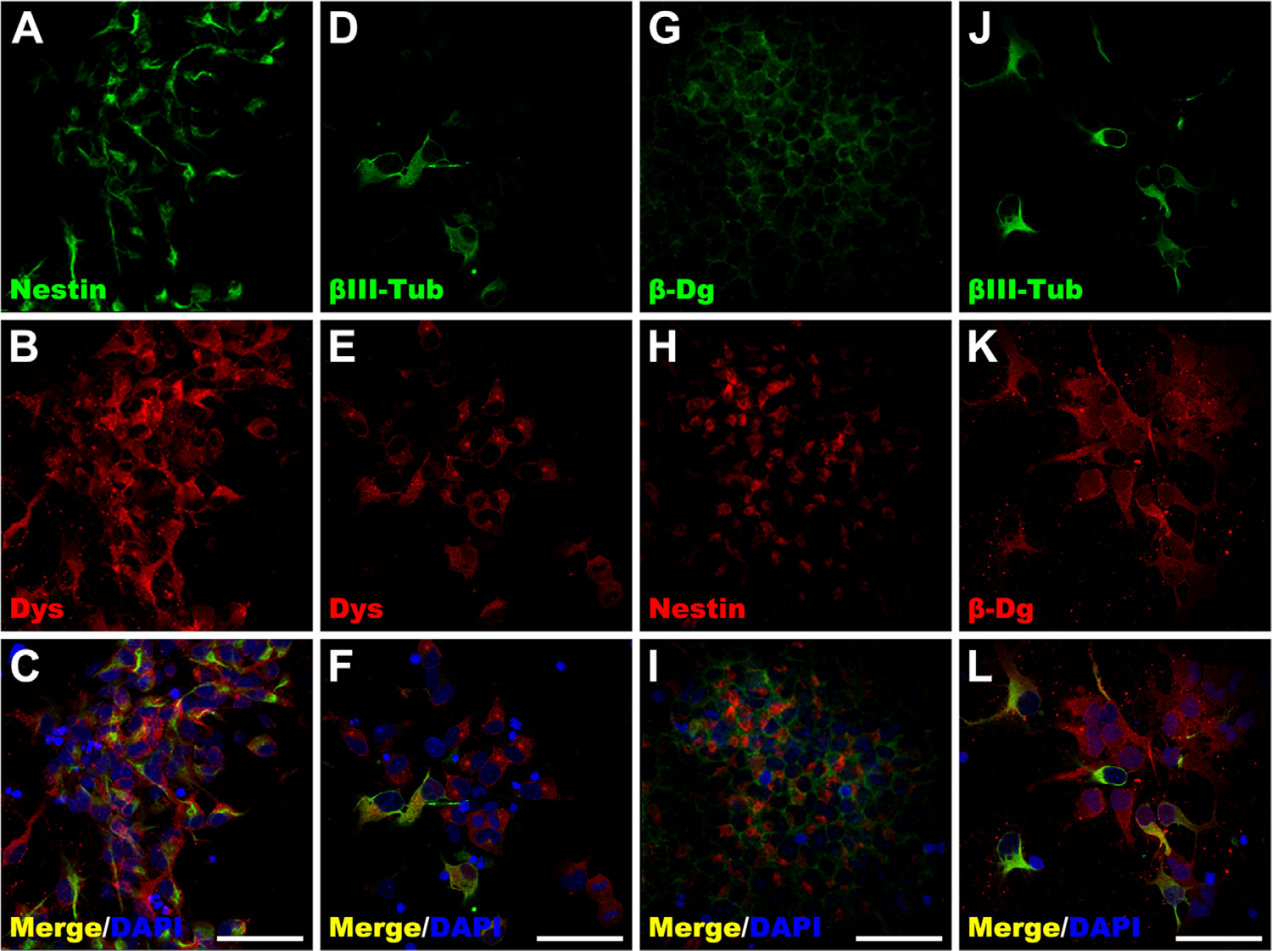
Subcellular localization of dystrophins and β-dystroglycan in undifferentiated NSPC. Undifferentiated NSPC at P0 were double immunostained with the antibodies: (A) anti-nestin mAb, (B, E) H4 (anti-dystrophins pAb) (Dys), (D, J) anti-βIII-tubulin mAb, (G) anti-β-dystroglycan mAb (β-Dg), (H) anti-nestin pAb, (K) JAF (anti-β-dystroglycan pAb) (β-Dg). Cells were subjected to analysis by confocal laser scanning microscopy and representative single confocal layers were selected in each case to show the subcellular distribution of the different proteins. All the right-hand panels (C, F, I, L) are merged images of the two preceding images. Nuclei were stained with DAPI. Scale bar corresponds to 50 µm. The results are representative of three independent experiments.

Neural stem/progenitor cells (NSPC) can be isolated from several brain regions and maintained in an undifferentiated state *in vitro* with mitogenic factors, such as basic fibroblast growth factor (FGF2). The FGF2 deprivation induces the differentiation of NSPC into neurons, astrocytes and oligodendrocytes [1], while the addition of histamine [2] or ciliary neurotrophic factor (CNTF) [3] promotes neuronal or glial differentiation, respectively. Here, we reported the isolation, culture and characterization of embryonic NSPC as a model to explore the expression and function of dystrophins and dystrophin-associated proteins (DAPs) during the neuronal or astrocytic differentiation. NSPC were differentiated with FGF2 deprivation and histamine or CNTF treatment. The expression of nestin as a multipotent marker, βIII-tubulin as a neuronal marker and GFAP as an astrocytic marker, was evaluated (Fig. 1). NSPC were also characterized by immunostaining for nestin, βIII-tubulin, dystrophins and β-dystroglycan (Fig. 2). Additionally, expression of dystrophins and dystrophin-associated proteins complex (DAPC) in proliferating and differentiating NSPC was characterized [4].

## Method details

### Animals

Adult female Wistar rats at 7 to 9 weeks-old and at least 200 g weight in oestrus were mated for 24 h with male Wistar rats at UPEAL- (Unit for Production and Experimentation of Laboratory Animals) Cinvestav. Animals were cared and handled following the guidelines for the use of laboratory animals provided by the CICUAL- (Internal Committee for the Care and Use of Laboratory Animals) Cinvestav and Mexican Official Norm (NOM-062-ZOO-1999).

### Isolation and culture of embryonic NSPC

On the 14th day, pregnant female rats were sacrificed via decapitation and embryos E14 were isolated, washed and maintained in ice-cold Krebs buffer (NaCl 100 mM, KCl 2 mM, KH_2_PO_4_ 600 nM, NaHCO_3_ 12 mM, Glucose 7 mM, MgSO_4_ 0.3%, BSA 0.3% and phenol red 0.1%, pH 7.4). The dorsal region of telencephalic vesicles was dissected with stereomicroscopic aid, and the isolation of the NSPC was performed as previously described [1], [2], [3]. Briefly, the tissue was mechanically dissociated in ice-cold Krebs buffer to a single cell suspension, and cells were recovered by centrifugation at 800–1000 *x g* for 3–5 min and were resuspended in N2 medium (DMEM/F12 1:1, supplemented with 25 mg/l human insulin, 30 nM sodium selenite, 100 μM putrescine, 20 nM progesterone and 100 mg/l Apotransferrin). Cells were seeded at 6 × 10^5^ in 6-cm diameter culture dishes previously treated with 15 µg/ml poly-L-ornithine (Sigma), and 1 µg/ml human fibronectin (Invitrogen) in N2 medium or Neurocult medium (Stem cell technologies) containing 10 ng/ml FGF2 (Peprotech). FGF2 was added daily, and the medium was changed every other day. The initial plating was considered passage 0 (P0), and when cells reached 80% confluence, they were detached and re-plated to P1 and, subsequently, to P2 to obtain a homogeneous cell population. P2 cells were maintained for 4 days in the control (N2 medium + 10 ng/ml FGF2) and experimental conditions (N2 medium + 10 ng/ml FGF2 with 100 µM histamine or 10 ng/ml CNTF). Then, differentiation was induced by FGF2 withdrawal, in the presence of 200 µM ascorbic acid as an antioxidant to avoid cell death added every three days, and maintaining histamine or CNTF treatment, which was added daily.

### RNA extraction and semi-quantitative RT-PCR

Total RNA was extracted using TRIzol (Invitrogen). Six to eight micrograms of RNA, treated with RNase-free DNase1, was retro-transcribed into cDNA using random hexamers (Invitrogen) and M-MLV reverse transcriptase (Promega), according to the manufacturer's instructions. cDNAs were amplified by PCR in a 25 µl reaction volume containing 0.5 µg cDNA and specific primers (Table 1). The PCR conditions comprised an initial denaturation at 94 °C for 5 min, followed by 18–30 cycles at 94 °C for 30 s, 57–65 °C for 30 s and 72 °C for 60 s, and a final extension at 72 °C for 5 min. PCR products were visualized by electrophoresis on 1.5–2.0% agarose-TAE gels, stained with ethidium bromide and analyzed with Kodak Digital Science software (Kodak). The number of cycles was determined within the linear range of amplification of each cDNA.

**Table 1:** Primer sequences and product sizes for the PCR reactions.

mRNA detected	Sequence 5’ to 3´		bp product size
	Forward	Reverse	
βIII-tubulin	GCCTCCTCTCACAAGTATGTGCC	AAGCCCTGCAGGCAGTCACAA	239
GFAP	GGGCGAAGAAAACCGCATCACC	TGGATGGGAATTGGGCCTAGCA	279
Nestin	GCAGCAACTGGCACACCTCAA	GCTTCAGCTTGGGGTCCAGAAA	143
MAP2	GCCTTTGGGGAGCACGGGTC	CCATCTTCGAGGCTTCTTCCAGTG	298
Sox2	CATTACCCGCAGCAAAATGACAGC	GAACTCCCTGCGAAGCGCCTA	172
β-actin	TTGTAACCAACTGGGACGATATGG	GATCTTGATCTTCATGGTGCTAGG	763

### Antibodies

H4 is a rabbit polyclonal antibody (pAb) that recognizes exon 78 of all dystrophin gene products, and JAF is a pAb antibody that recognizes β-dystroglycan. Both antibodies were kindly provided by Dr. Dominique Mornet and were previously characterized [5], [6], [7]. Anti-β-actin is a monoclonal antibody (mAb) kindly supplied by Dr. Manuel Hernández. All other antibodies were commercially available: mAb anti-dystroglycan (Ab49515, Abcam), mAb anti-βIII-tubulin (MMS-435P, Covance), pAb anti-nestin (PRB-315C, Covance), mAb anti-nestin (MAB2736, Developmental Studies Hybridome Bank), and pAb anti-GFAP (Z0334, DAKO). These antibodies were used in this data article, while the following antibodies: α1CT-FP, a pAb for α-dystrobrevin kindly provided by Blake et al. [8], mAb anti-dystrophins (Dys2) (NCL-DYS2, Novocastra), mAb anti-Sox2 (MAB2018, R&D), mAb anti-GFAP (MMS-588S, Covance), and mAb anti-microtubule-associated protein 2 (MAP2) (AB11267, Covance) were used in the principal article [4].

### Western blot analysis

NSPC protein extracts were prepared in extraction buffer (250 mM Tris, pH 8.0, and 1 mM EDTA with 1X complete protease inhibitors cocktail from Roche Inc.). Cells were sonicated, and protein concentrations were determined by spectrophotometry at 280 nm or by Bradford's method. The total extract was resuspended in sample buffer (75 mM Tris-HCl, 15% sodium dodecyl sulphate (SDS), 5% β-mercaptoethanol, 20% glycerol, and 0.001% Bromophenol Blue). Forty to sixty µg of protein was size fractioned on 8% or 10% polyacrylamide/SDS gels and were electrotransferred onto nitrocellulose membranes. Membranes were blocked for 2 h with fat-free milk solution, and the incubation for the primary antibodies pAb anti-nestin (1:500), mAb anti-βIII-tubulin (1:3000), pAb anti-GFAP (1:1500), and mAb anti-β-actin (1:2000) was to 24 h at 4 °C, followed by a 1 h incubation with the peroxidase-conjugated secondary antibody goat anti-rabbit IgG (A10547) or goat anti-mouse IgG (A10668) (1:10,000, Molecular Probes, Invitrogen). The detection of the immunoreactive bands was carried out using the Western Lightning ECL-Plus system (Perkin-Elmer, Inc.). The antibodies H4 (1:5000), JAF (1:5000), and α1CT-FP (1:1000) were used in the principal article [4].

### Immunofluorescence staining

NSPC were grown on coverslips pre-coated with poly-L-ornithine and fibronectin. Cells were fixed with 4% paraformaldehyde (PFA) for 2 min followed by 2% PFA for an additional 20 min, were washed three times with PBS and were blocked for 1 h with 10% normal goat serum (NGS) in PBS. Cells were incubated overnight at 4 °C with the primary antibodies diluted in PBS containing 10% NGS: pAb anti-nestin (1:1000), mAb anti-nestin (1:100), mAb anti-βIII-tubulin (1:2000), pAb H4 (1:20), pAb JAF (1:20), and mAb anti-dystroglycan (1:20), followed by a 1 h incubation with anti-mouse IgG Alexa Fluor 488 (A21202) and anti-rabbit IgG Alexa Fluor 594 (A21207) as secondary antibodies (1:200, Molecular Probes, Invitrogen). Nuclei were stained with DAPI (1 ng/ml, Sigma). Mounting was performed with Vectashield mounting medium (Vector laboratories, Inc.), and the samples were analyzed on a Leica TCS-SP8 confocal microscope. For each image, 10–15 optical z-sections (0.4 µm thick) were scanned. Representative sections were chosen to obtain the distribution of the different proteins in the NSPC. The images were analyzed using the Leica Application Suite AF software (Leica). The mAb anti-Sox2 (1:200), mAb anti-MAP2 (1:500), pAb anti-GFAP (1:2000), and mAb anti-GFAP (1:500) were used in the principal article [4].

### Statistical analysis

Statistical analyses were carried out with the GraphPad Prism 5 software. Data from three independent experiments were analyzed using unpaired Student t-tests. Statistical significance was considered when the *P* value was < 0.05.

## Additional information

### Expression of nestin, βIII-tubulin and GFAP during NSPC differentiation

Cells from the developing rat telencephalic vesicles were dissociated and kept in culture with FGF2. During the first passage, the cortical cerebral cell cultures contained a mixture of neural stem cells with progenitors and immature neurons, and in the subsequent passages, the proportion of NSPC increased. The undifferentiated state of NSPC can be verified with molecular markers, such as the transcriptional regulator Sox2, the intermediate filament protein nestin and/or surface antigens, such as Lex [9], [10], [11], [12]. P2 NSPC were maintained in an undifferentiated state by adding FGF2 and differentiation was induced by FGF2 withdrawal and histamine or CNTF treatment. To determine the nature of isolated NSPC, we analyzed the expression of multipotent (nestin and Sox2) and differentiation markers, with βIII-tubulin as a neuronal marker and GFAP as an astrocytic marker. As expected, the level of nestin mRNA was significantly down-regulated, whereas the levels of βIII-tubulin and GFAP mRNA were up-regulated upon FGF2 withdrawal and histamine or CNTF treatment (Fig. 1A), with similar results obtained at the protein level (Fig. 1B). Proliferating NSPC at P2 were also immuno-positive for both nestin and Sox2 [4] and were immuno-negative for βIII-tubulin and GFAP (data not shown), while differentiated cells were positive for βIII-tubulin, MAP2 and GFAP [4].

### Subcellular localization of dystrophins and β-dystroglycan in undifferentiated NSPC

As part of the characterization of NSPC, subcellular localization of dystrophins and β-dystroglycan as well as nestin and βIII-tubulin was analyzed on P0 and P1 cells. At P0, the level of nestin-immuno-positive cells reached 87% ± 10.6, while βIII-tubulin reached 17.3% ± 8.2 (Fig. 2D and J). As expected, no GFAP-positive cells were observed (data not shown). Interestingly, most of the nestin-positive cells were also positive for dystrophins (92.2% ± 11. 2), which were localized in the cytoplasm and cellular periphery, and in some cells, a slight staining was also observed in the nucleus (Fig. 2B and C). In addition, most of the βIII-tubulin-positive cells presented 99% ± 1.6 immunopositivity for dystrophins with two cell populations, one of which had predominant cytoplasm and cell periphery staining, and the other, which had cytoplasmic, cell periphery and nuclear localization (Fig. 2E and F). However, we were unable to determine whether such a differential subcellular distribution was related to the differences in specific dystrophin expression because the antibody used recognized exon 78, a common region for most of the dystrophin gene products. Regarding β-dystroglycan immunostaining, we observed that almost all the nestin-positive cells co-expressed β-dystroglycan (93.6% ± 2.5) and localized prominently in the cytoplasm and cellular periphery, and some cells showed clear nuclear staining (Fig. 2G and I). Similar to the dystrophin staining, most of the βIII-tubulin-positive cells co-expressed β-dystroglycan (98.9% ± 1.4), with two cell populations being observed: the first demonstrated β-dystroglycan localization principally in the cytoplasm and cell periphery, and the second having β-dystroglycan localization in the cytoplasm, cell periphery and nucleus (Fig. 2K and L).

At P1, the level of nestin-positive cells reached 90% ± 6.4 and that of βIII-tubulin reached 12.5% ± 3.3, while no GFAP-positive cells were found (data not shown). The subcellular localization of dystrophins and β-dystroglycan was basically the same as that at P0, and nestin-positive cells co-expressed dystrophins (99% ± 1.54) and β-dystroglycan (94.5% ± 8.7). βIII-tubulin-positive cells co-expressed dystrophins (99.6% ± 0.3) and β-dystroglycan (95.6% ± 7.6). Two populations of βIII-tubulin-positive cells were recognized: the first demonstrated subcellular localization of dystrophins and β-dystroglycan in the cytoplasm and cellular periphery, and the second demonstrated cytoplasmic, cellular periphery and nuclear staining (data not shown). In addition, subcellular localization of dystrophins and β-dystroglycan was evaluated at P2 and in differentiated NSPC. Interestingly, localization of dystrophins using Dys2 antibody was observed in the cytoplasm, cellular periphery, and in the nucleus as it was detected with H4 antibody [4].

## Declaration of Competing Interest

The authors declare that they have no known competing financial interests or personal relationships that could have appeared to influence the work reported in this paper.
